# Efficacy of Antibacterial Peptides Against Peptide-Resistant MRSA Is Restored by Permeabilization of Bacteria Membranes

**DOI:** 10.3389/fmicb.2016.01745

**Published:** 2016-11-08

**Authors:** Joshua Ravensdale, Zachary Wong, Frances O’Brien, Keith Gregg

**Affiliations:** ^1^School of Biomedical Science, Faculty of Health and Curtin Health Innovation Research Institute, Curtin University, BentleyWA, Australia; ^2^Australian Collaborative Centre for Enterococcal and Staphylococcal Species (ACCESS) Typing and Research, Curtin University and PathWest Microbiology, Royal Perth Hospital, PerthWA, Australia

**Keywords:** MRSA, antimicrobial peptides resistance, Melittin, Bac8c, intracellular antimicrobial activity

## Abstract

Clinical application of antimicrobial peptides (AMPs), as with conventional antibiotics, may be compromised by the development of bacterial resistance. This study investigated AMP resistance in methicillin resistant *Staphylococcus aureus*, including aspects related to the resilience of the resistant bacteria toward the peptides, the stability of resistance when selection pressures are removed, and whether resistance can be overcome by using the peptides with other membrane-permeabilising agents. Genotypically variant strains of *S. aureus* became equally resistant to the antibacterial peptides melittin and bac8c when grown in sub-lethal concentrations. Subculture of a melittin-resistant strain without melittin for 8 days lowered the minimal lethal concentration of the peptide from 170 μg ml^-1^ to 30 μg ml^-1^. Growth for 24 h in 12 μg ml^-1^ melittin restored the MLC to 100 μg ml^-1^. Flow cytometry analysis of cationic fluorophore binding to melittin-naïve and melittin-resistant bacteria revealed that resistance coincided with decreased binding of cationic molecules, suggesting a reduction in nett negative charge on the membrane. Melittin was haemolytic at low concentrations but the truncated analog of melittin, mel12-26, was confirmed to lack haemolytic activity. Although a previous report found that mel12-26 retained full bactericidal activity, we found it to lack significant activity when added to culture medium. However, electroporation in the presence of 50 μg ml^-1^ of mel12-26, killed 99.3% of the bacteria. Similarly, using a low concentration of the non-ionic detergent Triton X-100 to permeabilize bacteria to mel12-26 markedly increased its bactericidal activity. The observation that bactericidal activity of the non-membranolytic peptide mel12-26 was enhanced when the bacterial membrane was permeablized by detergents or electroporation, suggests that its principal mechanism in reducing bacterial survival may be through interaction with intracellular organelles or processes. Additionally, our results showed that the haemolytic peptide bac8c, had increased antibacterial activity at non-haemolytic concentrations when used with membrane-permeabilizing surfactants.

## Introduction

Antimicrobial peptides (AMPs) have been proposed as possible additions to, or replacements for, conventional antibiotics which are declining in efficacy due to increasing resistance among clinically important pathogenic bacteria (Yeaman and Yount, 2003; Reddy et al., 2004). They are generally amphipathic protein molecules of approximately 8-50 amino acids, usually have a net positive charge and contain a high proportion of hydrophobic residues (Yeaman and Yount, 2003; Reddy et al., 2004). AMPs appear to be a significant part of the innate immune system of many organisms, causing non-specific inhibition or destruction of bacterial pathogens, which appears to be mediated by peptides binding to anionic groups on the cell surface and interacting hydrophobically with membranes to form lethal pores (Gordon et al., 2005). Targeting the fundamental anionic and hydrophobic regions of bacterial membranes has been suggested to make the development of resistance toward cationic peptides unlikely, requiring significant alteration to the physiology of the cell (Gordon et al., 2005; Seo et al., 2012; Gaspar et al., 2013). Nevertheless, pathogens have been regularly exposed to AMPs and resistance or avoidance mechanisms have been described (Gordon et al., 2005; Wiesner and Vilcinskas, 2010).

Although there have been studies into how pathogens may develop resistance to AMPs (Andrä et al., 2011; Askoura et al., 2011; Guilhelmelli et al., 2013), it remains unclear how significant the development of resistance may be for the clinical use of AMP-based therapies. Also, some AMPs have intracellular targets that may be more important for killing the pathogen than membrane disruption (Laverty et al., 2011). The declining efficacy of antibiotics provides a strong incentive to understand how AMPs might be applied for therapeutic purposes, but there are still gaps in our understanding of which peptides may have intracellular antibacterial activity and how they may be delivered to the bacterial cytoplasm.

We selected the bactericidal peptides melittin, isolated from honey bee venom (Kreil and Bachmayer, 1971; Raghuraman and Chattopadhyay, 2007), and bac8c, a truncated and modified analog of bovine neutrophil peptide (Spindler et al., 2011), as model peptides to test the kinetics of resistance development, the resilience of the resistant state, and whether bacterial resistance might be defeated in clinical treatment. In common with some other antibacterial peptides, the haemolytic capability of melittin may preclude its clinical use (Asthana et al., 2004; Raghuraman and Chattopadhyay, 2007). However, a sub-fragment containing residues 12-26 of melittin has been reported to maintain antibacterial activity with considerably reduced haemolytic capability (Yan et al., 2003). We investigated the potential for transporting melittin, mel12-26, or bac8c into MRSA to increase their bactericidal efficacy.

## Materials and Methods

### Bacterial Strains and Growth Conditions

*Staphylococcus aureus* strains were provided by the Australian Collaborating Centre for *Enterococcus* and *Staphylococcus* Species typing and research (ACCESS), Faculty of Health Sciences, School of Biomedical Sciences, Curtin University, Perth, WA, Australia. The *S. aureus* strains used in this study were: Aus3: a mercuric acetate and mercuric chloride-resistant clone of ST239-MRSA-III (Coombs et al., 2007), Bengal bay (BB): origin ST772-MRSA-V (Coombs G. et al., 2012), and WBG 8287: genetic lineage ST1-IVa (Coombs et al., 2007) and a methicillin sensitive *S. aureus* strain W17S: lineage ST93-MSSA (Coombs G.W. et al., 2012). Bacteria were grown in 25 g l^-1^ bactopeptone medium (Oxoid, Basingstoke, Hants, UK), pH 7.4 and incubated at 37°C with orbital shaking at 200 rpm. Bactopeptone medium was selected as a growth and test medium because it showed less inhibitory effect on the antimicrobial activity of the peptides than the more commonly used complex and nutrient-rich media we tested (data not shown). A relatively simple and dilute medium was believed to be appropriate because clinical treatment of topical infections would be expected to include initial cleansing of the infected region with dilute solutions prior to the application of antimicrobial agents, to maximize antibacterial function.

### Antibacterial Peptides and Measurement of Minimal Lethal Concentrations

Bac8c (RIWVIWRR-OH), melittin (GIGAVLKVLTT**GLPALISWIKRKRQQ-OH**) and its sub-fragment mel12-26 (bold type) were in L-isomer form and 95% pure by mass spectrometry (Mimotopes, Melbourne, VIC, Australia). Peptides were dissolved in molecular biology grade water and tested for their minimal lethal concentration (MLC) against *S. aureus* strains, using a variation of a previously described method (Dean et al., 2011). Briefly, 200-μl aliquots of bacterial culture (10^6^ CFU ml^-1^ in 8 g l^-1^ bactopeptone) were transferred to a polystyrene 96-well plate (Nunc, New York, USA) containing the test peptides at final concentrations from 1 to 170 μg ml^-1^. The plates were incubated at 37°C with 200 rpm orbital shaking for 1 h, then bacteria from each well were plated on separate 1.5% agar plates, containing 25 g l^-1^ bactopeptone medium, and incubated at 37°C for 24 h. Survival was measured by colony numbers, as a percentage of untreated controls. Negative controls contained mock-inoculated medium. The lowest dose that resulted in >99.9% cell death was recorded as the MLC. Experiments were repeated two or three times, each with triplicate samples.

### Growth in Sub-inhibitory Doses of Melittin and Bac8c

Strains WBG 8287, W17S, Aus3, and BB were suspended at 10^7^ CFU ml^-1^ in 200 μl of 8 g l^-1^ bactopeptone broth, containing 2.5 μg ml^-1^ of melittin, and incubated for 12 h at 37°C with 150 rpm orbital shaking. Bacteria were counted and resuspended at 10^7^ CFU ml^-1^ in 200 μl of 8 g l^-1^ bactopeptone medium, containing 5 μg ml^-1^ melittin, and incubated for an additional 12 h. This was followed by subculture at six 12 h intervals, with melittin at 8, 10, 15, 20, 40, and 60 μg ml^-1^, respectively, and six more subcultures at 12 h intervals with 80 μg ml^-1^ melittin. Parallel experiments were performed with bac8c concentrations increasing from 2 μg ml^-1^ to 180 μg ml^-1^, for strains WBG 8287, W17S, and Aus3.

### Scanning Electron Microscopy of Bacteria Exposed to Melittin

Wild-type and melittin-resistant WBG 8287 were washed in 1 ml PBS (10^6^ CFU ml^-1^) and centrifuged at 12,000 × *g* for 5 min. The bacterial pellets were resuspended in 1 ml PBS, re-centrifuged, and suspended in 100 μl PBS containing 10 μg ml^-1^ of melittin for 1 h at 37°C with 200 rpm orbital shaking. Control samples were prepared by the same procedure, without melittin. Aliquots of each bacterial suspension (15 μl) were dispensed onto separate aluminum stubs and incubated for 30 min at 37°C. The bacteria were fixed by overlaying with 2.5% glutaraldehyde (Asia Pacific Specialty Chemicals Limited, Sydney, NSW, Australia) for 3 h at room temperature. The stubs were washed by gently applying high-purity water to the tilted surface, followed by sequential immersion for 30 min at 37°C in 70, 90, and 100% ethanol, respectively. The samples were dried in a desiccator for 24 h at 37°C over silica gel. The stubs were evaporatively coated with a 3 nm layer of platinum and viewed using a Zeiss Neon 40ESB Crossbeam scanning electron microscope (SEM: Sydney, NSW, Australia).

### Measurement of Cationic Fluorophore Binding to the MRSA Outer Surface, by Flow Cytometry

Wild-type and melittin-resistant WBG 8287 (10^7^ CFU ml^-1^) were washed twice in 1 ml phosphate buffer (PB) with centrifugation at 8,000 × *g* for 5 min and were resuspended in 1 ml of PB. A 100 μl aliquot of bacterial suspension was added to 400 μl of HpH_2_O (5 × 10^6^ CFU ml^-1^) containing 2.08 picomoles of the cationic fluorescent probe Bacterisense 645 (Perkin Elmer, Melbourne, VIC, Australia). The bacteria were then washed three times for 10 min each in PB, with shaking at room temperature and centrifugation, to remove unbound or weakly bound probe. Dye-binding to bacteria was measured using an Attune Acoustic Focusing Cytometer (Life Technologies, Melbourne, VIC, Australia) with Attune focusing fluid. For these experiments, all solutions including growth media were filtered through 0.2 μm membrane filters.

### Removal and Return of Melittin Selection

Melittin-resistant bacteria (WBG 8287) were subcultured daily for 7 days in bactopeptone medium without melittin. Their survival after exposure to 0, 3, 6, or 12 μg ml^-1^ melittin in 8 g l^-1^ bactopeptone medium for 1 h was measured daily. After seven days of melittin-free growth, the MLC of melittin was measured, the bacteria were returned to medium containing 12 μg ml^-1^ melittin for 24 h and the MLC was measured again.

### Preparation of Electro-Competent MRSA

Wild-type and melittin-resistant WBG 8287 (10^9^ CFU ml^-1^ in 1 ml PBS) were centrifuged at 8,000 × g for 10 min, washed with 500 mM sucrose, recentrifuged and resuspended in 500 μl of 500 mM sucrose. The bacteria were chilled on ice for 30 min, recentrifuged and resuspended in 500 μl of 500 mM sucrose with 15% glycerol and stored at -80°C. For use in electroporation experiments, frozen stocks were thawed at room temperature.

### Electroporation of MRSA with AMPs

Electro-competent wild-type WBG 8287 were placed in electroporation cuvettes (Gene pulser^TM^ Bio-rad, Sydney, NSW, Australia) with a 0.1 cm electrode gap, with melittin (2.5 μg ml^-1^) or without melittin (controls) and cuvettes were chilled on ice for 1 min. Bacteria were pulsed for 2.5 ms using a Micropulser electroporator (Bio-rad, Sydney, NSW, Australia) set at 25 μF capacitance, 2.5 kV and 100 Ω by-pass resistance. Immediately after electroporation, melittin was added to control samples at 2.5 μg ml^-1^ and 450 μl of 500 mM sucrose solution was added to all cuvettes. Bacterial suspensions were placed on ice for 15 min, spread on bactopeptone agar plates and incubated for 24 h at 37°C. Survival was measured by colony numbers as a percentage of a melittin negative, non-electroporated control. The same method was used to insert mel12-26 (50 μg ml^-1^) or bac8c (2 μg ml^-1^) into wild-type bacteria, and melittin (10 μg ml^-1^) or mel12-26 (50 μg ml^-1^) into melittin-resistant WBG 8287. Electroporation experiments were repeated three times, each with four replicates per peptide.

### Isolation of Human Erythrocytes and Haemolysis Assay

Whole blood (15 ml) was drawn from the vein of a 26 year old male volunteer and stored in an EDTA-coated Vacutainer (BD Biosciences, Adelaide, SA, Australia). Erythrocytes were separated by centrifuging at 500 × *g* for 5 min at 4°C. The supernatant was removed and erythrocytes were washed with 10 ml of ice-cold PBS and recentrifuged. Two otherwise identical sample groups were prepared by removing the supernatant and resuspending the erythrocytes in 10 ml PBS at 10^6^ cells ml^-1^, either with or without 0.005% v/v Triton X-100 (Ajax, Perth, WA, Australia). Melittin (5 μg ml^-1^), mel12-26 (130 μg ml^-1^) or bac8c (6 μg ml^-1^) was added to triplicate samples of PBS samples with no Triton X-100, and 2.5 μg ml^-1^ melittin, 80 μg ml^-1^ mel12-26, and 4 μg ml^-1^ bac8c were added to PBS samples with Triton X-100. Triton X100 was added to erythrocyte suspensions at a concentration of 1% v/v as a positive control for haemolysis. All test and control samples were incubated at 37°C for 30 min with 150 rpm orbital shaking, then sedimented at 500 × *g*. The extent of haemolysis was measured by the absorbance of the supernatant at 405 nm, using a Victor Multilabel plate reader spectrophotometer.

### Statistics

Data compared between parental and resistant bacterial populations were analyzed by the paired *t*-test using Microsoft Excel software.

## Results

### Minimum Inhibitory Concentration and Minimum Lethal Concentrations of AMPs for *S. aureus*

**Table 1** shows the MLC of antibacterial peptides for different *S. aureus* strains. The MIC and MLC of mel12-26 could not be determined because the concentrations required were beyond the solubility limit of the peptide in bactopeptone medium. Above 50 μg ml^-1^ the excess peptide remained as a cloudy and sedimentable suspension. The MLC of bac8c differed significantly in strain WGB 8287 (7 μg ml^-1^) from that of both W17S and Aus3 (both 80 μg ml^-1^).

**Table 1:** Minimum lethal concentration of melittin, mel12-26, and bac8c for MRSA strains in bactopeptone media.

Peptide	MRSA strain	MLC (μg ml^-1^)
Melittin	WBG 8287	5
	W17S	5
	Aus3	5
	BB	5
Mel12-26	WBG 8287	>130^a^
Bac8c	WBG 8287	7
	W17S	80
	Aus3	80


*^a^Minimal lethal concentration (MLC) exceeded the highest concentration tested.*

After 14 sub-cultures at 12 h intervals, in bactopeptone medium containing sub-inhibitory concentrations of melittin, the MLC for WBG 8287, W17S, Aus3, and BB was increased 30-34 fold (**Table 2**). While growth for 14 sub-cultures in the presence of bac8c increased the MLC of the peptide 23-fold for WBG 8287, only a twofold increase was required to reach similar levels of resistance in the naturally more resistant strains W17S and Aus3 (**Table 2**). The MLC for resistant WBG 8287 in bactopeptone broth was 170 μg ml^-1^, but in phosphate buffer was only 3 μg ml^-1^. The addition of NaCl at 8 and 15 g l^-1^ increased the MLC in phosphate buffer to 5 and 7 μg ml^-1^, respectively (**Table 2**), suggesting that salt concentration plays only a minimal role in peptide resistance.

**Table 2:** Minimal lethal concentration (MLC) of melittin and bac8c against MRSA in different media, following induced resistance to the peptides in bactopeptone medium.

MRSA Strain	Culture medium	Melittin MLC (μg ml^-1^)	Bac8c MLC (μg ml^-1^)
WBG 8287	Bactopeptone	170	160
	PB^a^	3	ND
	PB + 8 g l^-1^ NaCl	5	ND
	PB + 15 g l^-1^ NaCl	7	ND
W17S	Bactopeptone	150	170
Aus3	Bactopeptone	160	150
BB	Bactopeptone	150	ND


*^a^Phosphate buffer; ND, not determined.*

### Structural Appearance of WBG 8287 after AMP Exposure

**FIGURE 1:**
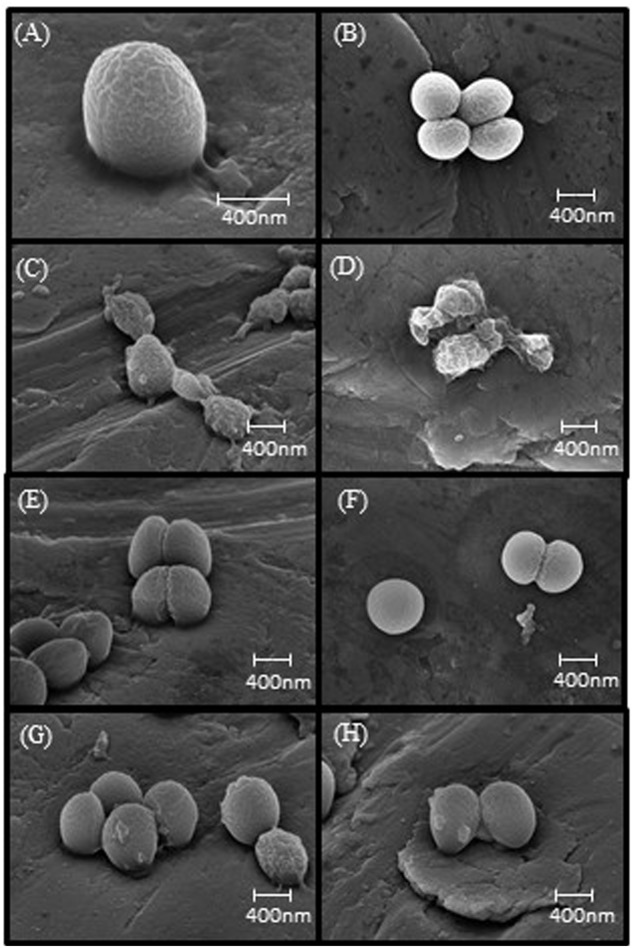
**Scanning electron microscopy images of melittin naïve and resistant MRSA.** **(A,B)** Untreated parental strain, **(E,F)** untreated melittin-resistant strain, **(C,D)** parental strain exposed to 10 μg ml^-1^ melittin. **(G,H)** Resistant strain exposed to 10 μg ml^-1^ melittin.

Scanning electron micrographs of control samples showed a rippled surface topography, which we interpret as showing the surface of the thin platinum coating over the peptidoglycan matrix of the cell wall (**Figures 1A,B**). Predictably, the development of melittin resistance caused no discernable changes in the external appearance of the cell wall (**Figures 1E,F**). After exposure to 10 μg ml^-1^ of melittin for 1h, wild-type strains showed major structural changes (**Figures 1C,D**) ranging from protrusions or “blebs” on the outer surface to the collapse of cellular structure. Under the same conditions, melittin-resistant WBG 8287 showed occasional surface blebs, but none displayed major loss of structural integrity (**Figures 1G,H**).

### Binding of Bacterisense 645 to Wild-type and Melittin-Resistant MRSA

**FIGURE 2:**
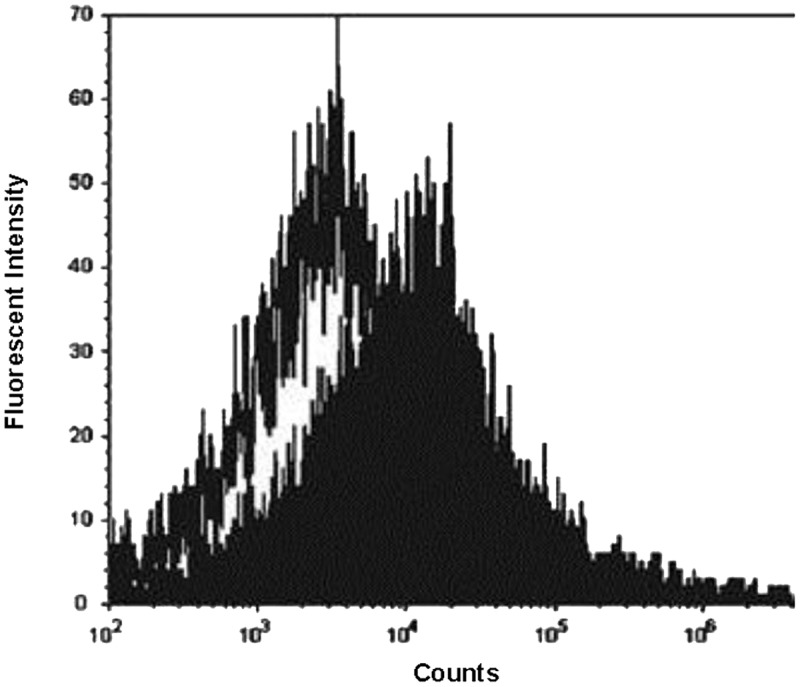
**Flow cytometry analysis of fluorescent probe binding to MRSA.** Fluorescence intensity of cationic fluorophore Bacterisense 645 bound to parental strain WBG 8287 (closed plot area) and melittin-resistant WBG 8287 (open plot area). Data represent the mean of three tests.

The mean fluorescent intensity emitted from the cationic probe Bacterisense 645, bound to the surface of wildtype and melittin resistant strains totalled 34099 and 6445, respectively (*P* < 0.001; **Figure 2**) suggesting that the negative charge responsible for binding the cationic fluorophore to the outer surface of melittin-resistant bacteria was only 19% of that present on the outer surface of the melittin-sensitive parental strain.

### Removal and Return of Melittin Selection

**FIGURE 3:**
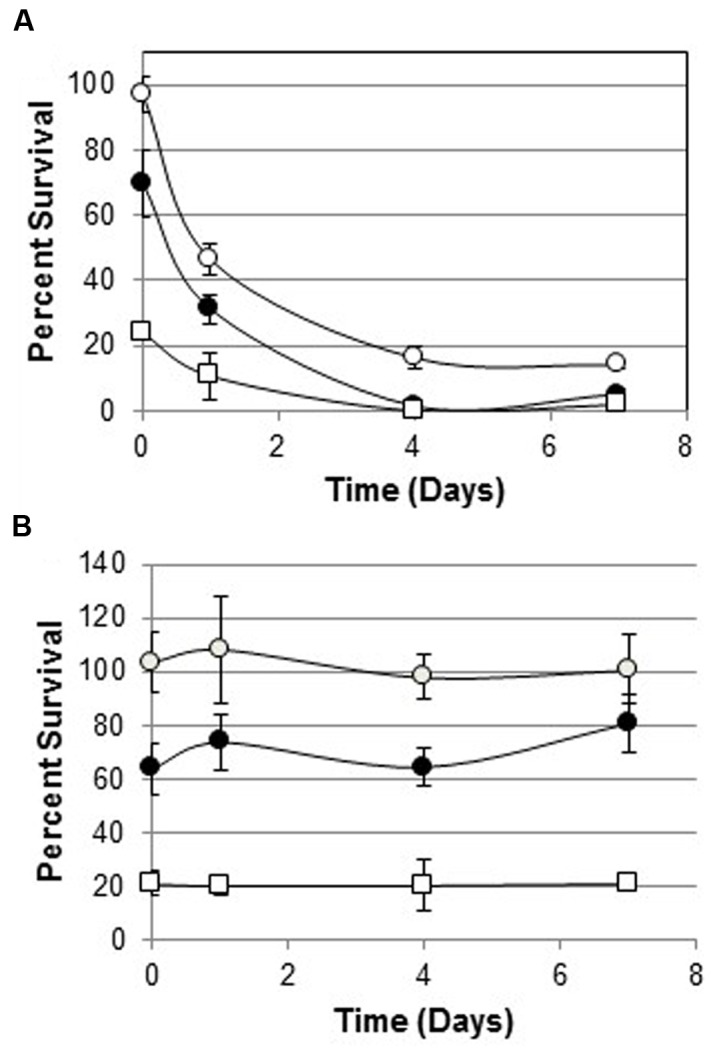
**Survival of melittin-resistant bacteria cultured with or without melittin for 7 days.** **(A)** Survival of parental WBG 8287 as a percentage of untreated controls, after exposure to melittin at 3 μg ml^-1^ (open circles), 6 μg ml^-1^ (closed circles), and 12 μg ml^-1^ (open squares) on days 0, 1, 4, and 7, respectively. **(B)** Survival of melittin resistant WBG 8287, maintained in medium containing 12 μg ml^-1^ melittin. Data represent combined results of two experiments with three replicates per experiment, Error bars represent standard deviation.

One hundred percent of melittin-resistant WBG 8287 survived when exposed to 3 μg ml^-1^ melittin in bactopeptone medium for 1 h. However, after growth for 24 h in bactopeptone medium without melittin, only 46% survived the same treatment. (*P* < 0.001; **Figure 3**). Eight daily subcultures without melittin, reduced the MLC of melittin from 170 μg ml^-1^ to 30 μg ml^-1^ (**Table 3**). However, a single 24 h culture of this resensitized population, in 12 μg ml^-1^ of melittin, raised the MLC to 100 μg ml^-1^: i.e., 58.8% of that seen in the most resistant population (**Table 3**).

**Table 3:** Minimum lethal concentrations of antimicrobial peptides (AMPs) for resistant WBG 8287 and reversion/recovery populations.

Population	MLC μg ml^-1^
Melittin-resistant	170
Melittin-reversion	30
Melittin-recovery	100
Bac8c-resistant	160
Bac8c-reversion	110


### Electroporation of MRSA in the Presence of AMPs

**FIGURE 4:**
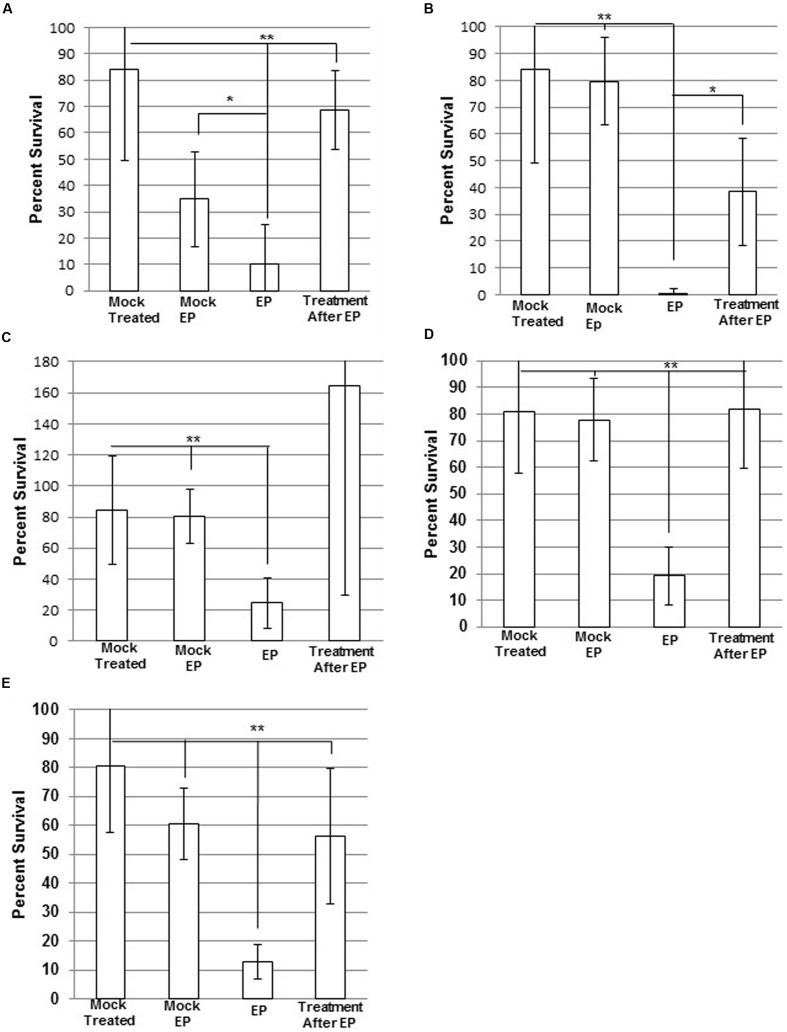
**Electroporation of MRSA with antimicrobial Peptides (AMPs).** Electrocompetent WBG 8287 cells suspended in **(A)** 2.5 μg ml^-1^ melittin, **(B)** 50 μg ml^-1^ mel12-26, and **(C)** 2 μg ml^-1^ bac8c and electroporated immediately (EP). Melittin-resistant WBG 8287 suspended in **(D)** 10 μg ml^-1^ melittin or **(E)** 50 μg ml^-1^ mel12-26 and electroporated. Survival was measured as a percentage of zero peptide, unelectroporated controls. Data represent the mean of six samples and error bars represent standard deviation. ^∗^*P* ≤ 0.05; ^∗∗^*P* ≤ 0.001.

WBG 8287 electroporated in 500 mM sucrose showed 78% survival, compared to mock-electroporated controls. Electroporation in the presence of antibacterial peptides resulted in significantly greater cell death than simple exposure to the same peptide concentration. Electroporation with melittin (2.5 μg ml^-1^) or mel12-26 (50 μg ml^-1^) reduced the number of surviving colonies to 75 and 21% of non-electroporated controls controls, respectively (*P* < 0.05; **Figures 4A–C**). Survival of control group bacteria, exposed to bac8c immediately after electroporation, was highly variable; with results falling outside the test required for significance (*P* = 0.053). Electroporation of melittin-resistant bacteria in the presence of 10 μg ml^-1^ of melittin (**Figure 4D**) or 50 μg ml^-1^ mel12-26 (**Figure 4E**) reduced survival to 41 and 52% of control values, respectively.

### Effects of Triton X-100 on AMP Activity

**FIGURE 5:**
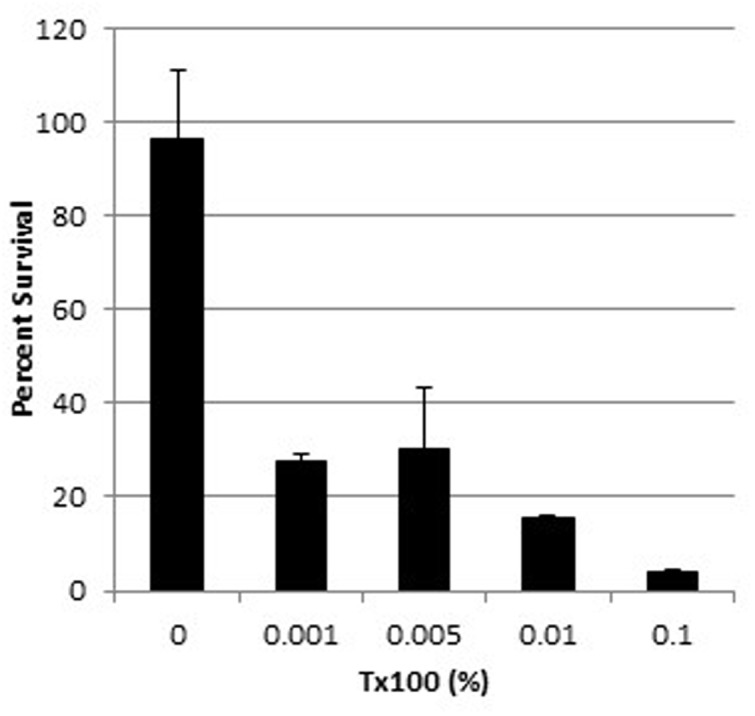
**Survival of WBG 8287 exposed to 5 μg ml^-1^ mel12-26 with increasing concentrations of Triton X-100.** Data represent the mean of three tests and error bars show standard deviation.

The survival of wild-type WBG 8287 exposed to mel12-26 decreased as the Triton X-100 concentration increased (**Figure 5**). The MLC of melittin (5.0 μg ml^-1^) decreased to 3.5 and 2.75 μg ml^-1^ in the presence of 0.005 and 0.1% Triton X-100, respectively (**Table 4**). In medium with 0.005 or 0.1% Triton X-100, the MLC of mel12-26 was 80 and 25 μg ml^-1^, respectively (**Table 4**). Similarly, increasing Triton X-100 concentration from 0.005 to 0.1% reduced the MLC of bac8c from 4.7 to 2.6 μg ml^-1^ (**Table 4**).

**Table 4:** Minimum lethal concentration of AMPs against WBG 8287 in bactopeptone with Triton X-100.

Peptide	BP^a^ (μg ml^-1^)	BP + 0.005% TX-100^b^ (μg ml^-1^)	BP + 0.1% TX-100 (μg ml^-1^)
Melittin	5	3.5 ± 0.55	2.75 ± 0.27
Mel12-26	>130	80 ± 0.00	25 ± 0.00
Bac8c	7	4.7 ± 0.52	2.6 ± 0.49


*^a^8 g l^*–1*^ bactopeptone; ^b^Triton X-100.*

The MLC of melittin or mel12-26 against melittin-resistant WBG 8287, W17S, and Aus3 bacteria was not affected by the presence of Triton X-100 at the non-haemolytic concentration of 0.005%.

### Haemolysis by Triton X-100 in Combination with AMPs

**FIGURE 6:**
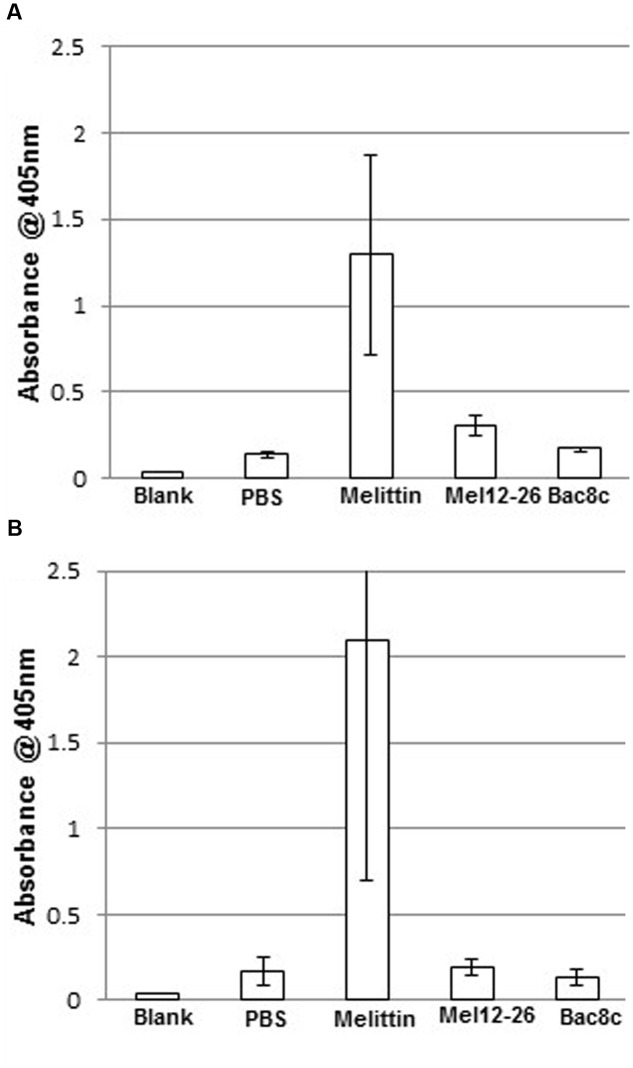
**Hemolysis by antibacterial peptides with Triton X-100.** Release of hemoglobin into the supernatant in **(A)** PBS with: melittin 5 μg ml^-1^, mel12-26 130 μg ml^-1^, or bac8c 6 μg ml^-1^. **(B)** PBS plus 0.005% Triton X-100 with: melittin 2.5 μg ml^-1^, mel12-26 80 μg ml^-1^, or bac8c 4 μg ml^-1^. Data represent the mean of three samples and error bars represent standard deviation.

In PBS, 5 μg ml^-1^ of melittin showed substantial erythrocyte lysis with or without 0.005% Triton X-100 (**Figure 6**). Suspension of erythrocytes in PBS plus 0.005% Triton X-100 (A_405_ = 6.4% of total lysis) differed only marginally from suspension in PBS alone (A_405_ = 5.1% of total lysis).

## Discussion

The rapidity with which resistance to melittin or bac8c is initiated and enhanced suggests that resistance mechanisms pre-exist within populations of *S. aureus*. The use of AMPs as antimicrobial agents could be impaired by the rapid development of resistant populations and further complicated by the effect of salts and exogenous proteins in reducing peptide toxicity (Lee et al., 1997; Hancock, 2000; Gordon et al., 2005; Brogden and Brogden, 2011; Lofton et al., 2013).

Scanning electron microscopy confirmed that resistant bacteria were visibly less damaged by exposure to melittin than the parental strain, as predicted from their enhanced survival. Nevertheless, protruding vesicles, or “blebs”, observed in this and previous studies (Hartmann et al., 2010), were visible on the surface of some resistant cells, suggesting that melittin resistance is manifested as a reduced degree of damage, rather than an all or none protection. Within the parental population, a very small proportion of cells showed less structural damage than the majority, suggesting that this resistant sub-group may restore and maintain population numbers during exposure to high concentrations of melittin.

On withdrawal of melittin from cultures, resistance declined rapidly but not completely. This differed from a previous study (Perron et al., 2006), which reported that resistance in *Pseudomonas fluorescens* and *E. coli* to pexiganan, an analog of magainin peptides from the skin of the African clawed frog, remained stable over 4 days without selective pressure. The rapid decline of resistance to melittin suggests that maintaining resistance may impose an increased metabolic demand. Some antibiotic and AMP-resistant bacteria have slower generation rates than wild-type bacteria or are unable to grow on nutrient depleted media (Lofton et al., 2013), which may represent the metabolic cost of resources used in developing and maintaining resistance to antimicrobial agents (Andersson and Hughes, 2010). However, multiple studies have shown some antibiotic-resistant bacteria have increased growth rates over their parental population in certain media (Björkman et al., 2000; Nagaev et al., 2001; Yu et al., 2005; Andersson and Hughes, 2010), and resistant bacteria can persist in patients as long as 1–4 years after treatment (Sjolund et al., 2003; Maria et al., 2005). This casts doubt on whether the metabolic cost of resistance must eventually cause a reversion of the population to its wild-type in every case, when selection pressures are removed. However, our study clearly observed reversion to relative peptide-sensitivity in these *S. aureus* strains.

Consistent with previous findings (Ouhara et al., 2008; Andrä et al., 2011), increased tolerance to melittin was attributable at least partly to a reduction in negative-charge density on the outer bacterial surface. It has been suggested that decreased affinity of cationic molecules for the bacterial outer membrane results in nature from aminoacylation of negatively charged phospholipid head-groups with cationic amino acids (Andrä et al., 2011). This hypothesis is consistent with a reduction in the outer membrane negative charge on peptide-resistant bacteria, as suggested by the reduced binding of the cationic probe Bacterisense 645. A proportion of the wild-type and resistant population appeared to overlap in the measured fluorescent intensity created by the binding of Bacterisense 645. Consistent with observations from the resistance-reversion experiments, this supports the proposal that the MRSA population maintains a range of levels of anionic surface charge. Since the starting cultures have no known prior exposure to AMPs, it appears that this may be an intrinsic population safety measure against cationic toxins encountered in nature. Likewise, it may be beneficial for population growth that not all of the bacteria in the resistant population keep their anionic charge-masking modifications, due to the increased metabolic demand of the process.

Melittin, mel12-26, and the unrelated peptide bac8c, showed increased bactericidal activity when inserted into WBG 8287 by electroporation. This was concluded to result from internalization of the peptides, rather than from membrane damage caused by electroporation, because controls electroporated without peptides showed significantly higher survival rates in every case. Control bacterial suspensions to which peptides were added immediately after electroporation showed significantly higher survival rates than those exposed to the peptides during electroporation. In contrast to an earlier report (Yan et al., 2003), in our hands the melittin sub-fragment mel12-26 showed low external toxicity to *S. aureus*, and the susceptibility levels observed more closely match those reported by Subbalakshmi et al. (1999). However, when transported into the cell by electroporation, mel12-26 showed a fourfold increase in antibacterial activity. Other studies (Asthana et al., 2004; Pandey et al., 2011) have suggested that the leucine zipper motif of melittin and similar peptides promotes dimerization and secondary structure formation. Truncating melittin, to retain only amino acids 12-26, removed the leucine zipper sequence and reduced its haemolytic capability. The absence of the zipper motif also significantly reduced extracellular bactericidal activity, while apparently retaining significant intracellular toxicity.

Bacteria resistant to the extracellular activity of melittin remained sensitive to the intracellular toxicity when peptides were internalized by electroporation, but were still measurably less sensitive to the intracellular activity of melittin or mel12-26 than wild-type bacteria. It can be hypothesized that this observation may also reflect some developed protection against internal toxic effects. Prolonged exposure to these AMPs may induce, or select for, mutations that enhance intracellular protection against melittin, or activate facultative protective mechanisms such as efflux pumps and peptidases. Previous studies (Hsu et al., 2005; Marchand et al., 2006) have shown that indolicidin can inhibit mRNA transcription by covalent bonding at specific DNA sequences. Although the intracellular targets of melittin have not yet been identified, the cationic charge and leucine zipper motifs of melittin may cause non-specific binding to nucleic acids (McCormick et al., 1996; Pavia et al., 2012).

Previous studies have shown that permeabilization of artificial membrane vesicles by three different lipo-peptide antibiotics (Patel et al., 2014) and the toxicity of oxacillin for MRSA (Komatsuzawa et al., 1995) were increased in the presence of a non-ionic detergent. Our study has shown a similar increase in the antibacterial activities of mel12-26, melittin, and bac8c against wild-type MRSA with increasing concentrations of Triton X-100. This suggests either that permeabilization of the bacterial outer membrane facilitates access for peptides to intracellular targets, or that the detergent enhances membrane instability. The relatively small increase in bactericidal activity of melittin and bac8c in the presence of Triton X-100 may reflect strong existing membrane penetrating forces that make the effects of other permeabilizing agents only marginal. The presence of Triton X-100 did not increase the bactericidal efficacy of melittin or mel12-26 against resistant bacteria. It is possible that outer surface modifications on resistant bacteria may reduce the cells’ sensitivity to other membrane permeabilizing agents, as shown in *Salmonella enterica* by McKelvey et al. (2014).

The relatively rapid gain and loss of peptide resistance described here is in stark contrast to the reported dynamics of antibiotic resistance stability over time (Andersson and Hughes, 2011). Our results suggest that the capability for development of resistance to an AMP may not preclude its therapeutic use in strategically designed regimens. Furthermore, the antibacterial efficacy of AMPs may be enhanced by combining them with membrane-surfactants.

## Author Contributions

JR was the principal researcher for the project, and was the primary author of the paper. KG and FO contributed significantly to the design and planning of the project, and ZW provided assistance with the acquisition of data. KG provided substantial assistance with the drafting, editing and submission of the manuscript. FO and ZW also made a significant contribution to the editing of the manuscript.

## Conflict of Interest Statement

The authors declare that the research was conducted in the absence of any commercial or financial relationships that could be construed as a potential conflict of interest.
